# Increasing physical activity in moderate-severe traumatic brain injury: protocol for a two-stage randomized controlled trial of a remote, mHealth-enhanced intervention

**DOI:** 10.3389/fresc.2026.1656326

**Published:** 2026-02-02

**Authors:** Tessa Hart, Monica Vaccaro, Lauren Krasucki, Inna Chervoneva, Amanda Rabinowitz

**Affiliations:** 1 Jefferson Moss Rehabilitation Research Institute, Elkins Park, PA, United States; 2 Division of Biostatistics, Thomas Jefferson University Sidney Kimmel Medical College, Philadelphia, PA, United States

**Keywords:** intervention, mobile health, physical activity, randomized controlled trial, traumatic brain injuries

## Abstract

**Objective:**

Protocol for randomized controlled trial (RCT) examining effects of novel, remotely delivered intervention (called GetUp&Go) to increase physical activity (PA) in chronic, moderate-severe traumatic brain injury (msTBI), including a mobile health (mHealth) component.

**Design:**

RCT (Clinicaltrials.gov NCT06028334) with 1:1 randomization to 10 weeks of immediate treatment (IT) or waitlist (WL), with primary outcome measured at 10 weeks. A second randomization to 10 weeks of continued mHealth support vs. no treatment will allow for examination of effects of mHealth on maintenance of treatment gains.

**Participants:**

70 community-dwelling adults ≥6 months post msTBI; medically cleared and physically/ cognitively able to participate; physically inactive (≤23 weekly moderate/ vigorous activity units on Godin Leisure-Time Exercise Questionnaire).

**Interventions:**

10-week GetUp&Go program: manualized, remotely delivered intervention with ingredients based on theoretical model of behavior change, in which participants set individual goals and programs for increasing PA; mHealth support via chatbot that delivers personalized messages, reminders, and reinforcement to participant phone.

**Main outcome measures:**

Primary outcome is activity count measured by accelerometer worn on wrist for 7 days at all assessment intervals. Secondary outcomes include emotional function, fatigue, sleep, pain, health-related quality of life.

**Discussion:**

While conclusions await the results of the trial, we consider PA enhancement to be a valuable and under-studied direction for treatment of msTBI. The advantages of the described treatment include strong theoretical and empirical basis for the treatment protocol, which has been designed to help to circumvent difficulties with initiation, persistence, and memory that interfere with the ability to develop healthful habits and routines following msTBI.

## 1 Introduction

The importance of physical activity (PA) for human health and well-being is incontrovertible. Widely cited guidelines state that adults aged 18+ should engage weekly in at least 150 min of moderate-to-vigorous PA (MVPA) or 75 min of vigorous-intensity PA, plus ≥2 bouts of strengthening activities involving all major muscle groups (1). PA at this level is associated with many health benefits including lower risk of all-cause mortality, reduced risk of depression and anxiety, improved sleep, and better quality of life (2). In the general population there is also a strong association between PA and emotional well-being (3).

Moderate to severe traumatic brain injury (msTBI) is a significant public health problem. It is estimated that in the US alone, almost 20% of the population has experienced TBI with loss of consciousness (4). Although many TBIs lead to transient symptoms, msTBI can cause potentially lifelong difficulties with physical, cognitive, and emotional function, leading to the recent conceptualization of TBI as a chronic health condition (5). A growing body of research shows that people with msTBI have decreased PA levels after msTBI compared to preinjury (6–8), as well as increased propensity for sedentary behavior and weight gain (9) and significantly elevated risk of cardiovascular disease (10). Among the many barriers to PA reported by those with msTBI are potential for pain, discomfort, or embarrassment; safety concerns; insufficient knowledge about PA or lack of access to PA-friendly spaces; limitations in time, transportation and other resources; fatigue; and poor motivation (11–13).

Despite these barriers, people with msTBI have expressed the view that PA is very important and report a high level of interest in programs promoting PA (12, 14), particularly those adapted to the specific needs of people with brain injury (15). PA programs delivered remotely are also acceptable to this population (16). Studies show that even those with mobility limitations due to msTBI are able to attain cardiovascular fitness equivalent to their uninjured counterparts (17). Several reports suggest that PA also improves cognitive function following msTBI (18, 19). An intriguing observational study (20) revealed that in persons with a history of TBI, there were significantly stronger correlations between PA and self-reported measures of cognitive function and global health, compared to the same correlations in uninjured controls. Although causality cannot be assumed, one implication of this finding is that people with TBI could experience a particularly high degree of health benefit from increased PA.

There is a modest literature describing efforts to increase PA for people with msTBI; most have tested short-term aerobic exercise programs, using a variety of outcome measures. A 2020 systematic review included 9 studies that used reduction in depression symptoms as a primary outcome (21). Small to medium positive effects on mood were noted overall; however, the effects on sustained PA were largely unexamined. Another group of studies examined cardiovascular endurance and/ or resistance training. A 2010 narrative review (22) and a 2017 Cochrane review (23) concluded that exercise training is safe in msTBI and mostly effective in improving fitness, but that the clinical value remained unclear. That is, the impact on metabolic measures of fitness was confirmed, but not the effect of increased PA in daily life or health and well-being. A more flexible approach, using individually tailored PA plans rather than prescribed exercise regimens, has been used by a few investigators. For example, Clanchy and colleagues (24, 25) delivered 10 sessions of PA promotion to persons with msTBI and other forms of acquired brain injury, both in-person and by phone, with the frequency of contact diminishing over time. The program emphasized safe, sustainable, and enjoyable activities, and included individualized relapse-prevention strategies as a final step. Therapist contact was withdrawn so that self-maintained PA could be measured 12 weeks after treatment cessation. While robust effects were found for increased PA after treatment, there was limited maintenance of gains at follow-up (24). The authors emphasized the importance of continued support to maintain gains in PA. Similarly, the most recent systematic review of PA interventions for msTBI (26) echoed the need for follow-up. This review also noted that the majority of studies to date are of poor methodologic quality, limiting conclusions about the impact of PA on function and quality of life in this population. The authors also urged the use of a broader range of outcome measures, including health and participation measures; more attention to study power; and more rigorous study design.

As detailed below, we have attended to all of these recommendations in developing the current trial. We designed an intervention for promoting PA in people with msTBI that has the following characteristics.

### 1.1 Theoretical basis

Few efforts to increase PA in this population have been grounded in theory, yet the literature on PA promotion in the general population has yielded rich insights from theoretical models of health-related behavior change. The ingredients used in the current intervention were derived from a highly influential model: the COM-B framework of Michie and colleagues (27). This framework, which has been called a “meta-theory (28),” has been rigorously developed and validated in extensive research on health behavior (29–33) and was strongly supported in a review of major theoretical approaches to PA promotion (34). According to this model, voluntary behavior and changes in behavior are a function of three factors: **C**apability, **O**pportunity, and **M**otivation. Capability refers to both cognitive/ psychological capacity, e.g., knowledge about why and how to perform a desired behavior and remembering to do it, and the physical ability to perform it. Opportunity means access to necessary social and physical/ logistic resources, including space, time, and social support. Motivation refers both to conscious/ reflective factors affecting drive and reward, and reflexive/ automatic motivation (e.g., priming) that may enhance habit formation (27).

### 1.2 Individual tailoring

Within this theoretical framework, we designed the intervention to accommodate a wide range of individual choices and physical capabilities. In this regard we were guided by the work of Clanchy et al. (24, 25) as well as research showing a *dose-response* relationship between PA increase and health benefit—meaning that it is not necessary to meet the standard recommendations for PA levels to experience improvements in mood, energy, and quality of life, as well as health risk reduction (35).

### 1.3 Remote delivery and mobile health support

Studies have revealed that personalized PA programs can successfully be delivered via remote means including phone, web, and text messages (36, 37). A recent review focused on smartphone-delivered PA promotion for mental health emphasized the importance of including education about the value of PA and personalized coaching within the intervention (38). There is a burgeoning literature showing that persons with msTBI, even those with significant neurocognitive impairment, can successfully engage in, and benefit from, interventions delivered via phone (39–41) and other remote methods. Mobile health (mHealth) interventions, such as smartphone apps and interventions delivered by text, are increasingly being used in TBI rehabilitation and research (42, 43). As described below, the current study provides both remote therapist contact and mHealth support from a chatbot called RehaBot, which was developed in our laboratory specifically for the needs of participants with TBI. In a feasibility study, participants with msTBI found RehaBot both easy to use and enjoyable (44).

### 1.4 Objective measurement of PA

There is considerable evidence that self-report measures of PA are less reliable than objective measurement using accelerometers worn on the body (45); the latter method has been shown to be reliable in persons with TBI (46). In this study, our primary outcome is increase in PA from pre- to post-treatment, measured with a wrist-worn accelerometer that has been successfully used with the msTBI population (47).

### 1.5 Attention to long-term impact

As described below, we include a follow-through phase to assess the effects of the intervention after therapist support is withdrawn, with randomization either to no continued support, or continued use of RehaBot (without therapist intervention). We also include treatment ingredients that have been shown to help sustain new PA habits, and less sedentary behavior, as part of daily life (36, 48).

### 1.6 Consumer input

Throughout intervention development, consumer input was obtained through multiple structured methods. Two paid consumer consultants with lived experience of msTBI and self-reported difficulties with PA were engaged in this process. Input was collected through facilitated focus group sessions where consultants reviewed and provided feedback on the treatment rationale and session structure; visual materials and participant handouts; and the use of RehaBot to deliver just-in-time support for PA in participants' daily environments. Additionally, consumer consultants participated in pilot testing of treatment sessions, providing feedback on feasibility, acceptability, and comprehensibility of intervention components. This iterative feedback was incorporated into the final treatment manual. Consultants also selected the name of the intervention: *GetUp&Go*.

### 1.7 Aims and hypotheses

The aim of this study is to examine the efficacy of the GetUp&Go intervention for increasing PA and enhancing associated functional outcomes in msTBI. We propose the following hypotheses:

1a. Participants who receive the GetUp&Go intervention directly after randomization will show significantly more increase in PA, as assessed by average accelerometer activity counts/ day, from pre-randomization baseline to post-treatment 10 weeks later, compared to those waitlisted for 10 weeks.

1b. Participants randomized to immediate treatment (IT) will also show significantly more improvement compared to waitlisted participants (WL) on secondary outcome measures including intensity of PA (derived from accelerometer readings), self-reported PA, emotional function, fatigue, sleep, pain, and health-related quality of life.

2a. Participants randomized to continued use of RehaBot for 10 weeks after treatment (RB) will demonstrate significantly less diminution in accelerometer-based activity counts from post-treatment to 10-week follow-up compared to those for whom RehaBot is withdrawn (No Tx), adjusted for the degree of change in the initial treatment phase.

2b. Participants randomized to RB in the follow-through phase will demonstrate significantly less diminution/ worsening in other outcome domains listed under Hypothesis 1b, compared to No Tx participants.

In addition to testing these hypotheses, we will explore the predictors of treatment response, using pooled data from all participants from pre- to post-treatment. We will analyze in exploratory fashion the predictive effects of demographic and injury characteristics; baseline cognitive, physical, social, emotional, and personality factors; objective and perceived neighborhood characteristics; intrapersonal variables such as motivation, self-efficacy, outcome expectancies, and identity/ values as related to PA; and process variables such as the amount of interaction with RehaBot during treatment.

## 2 Methods

### 2.1 Overview of design

**Figure 1:**
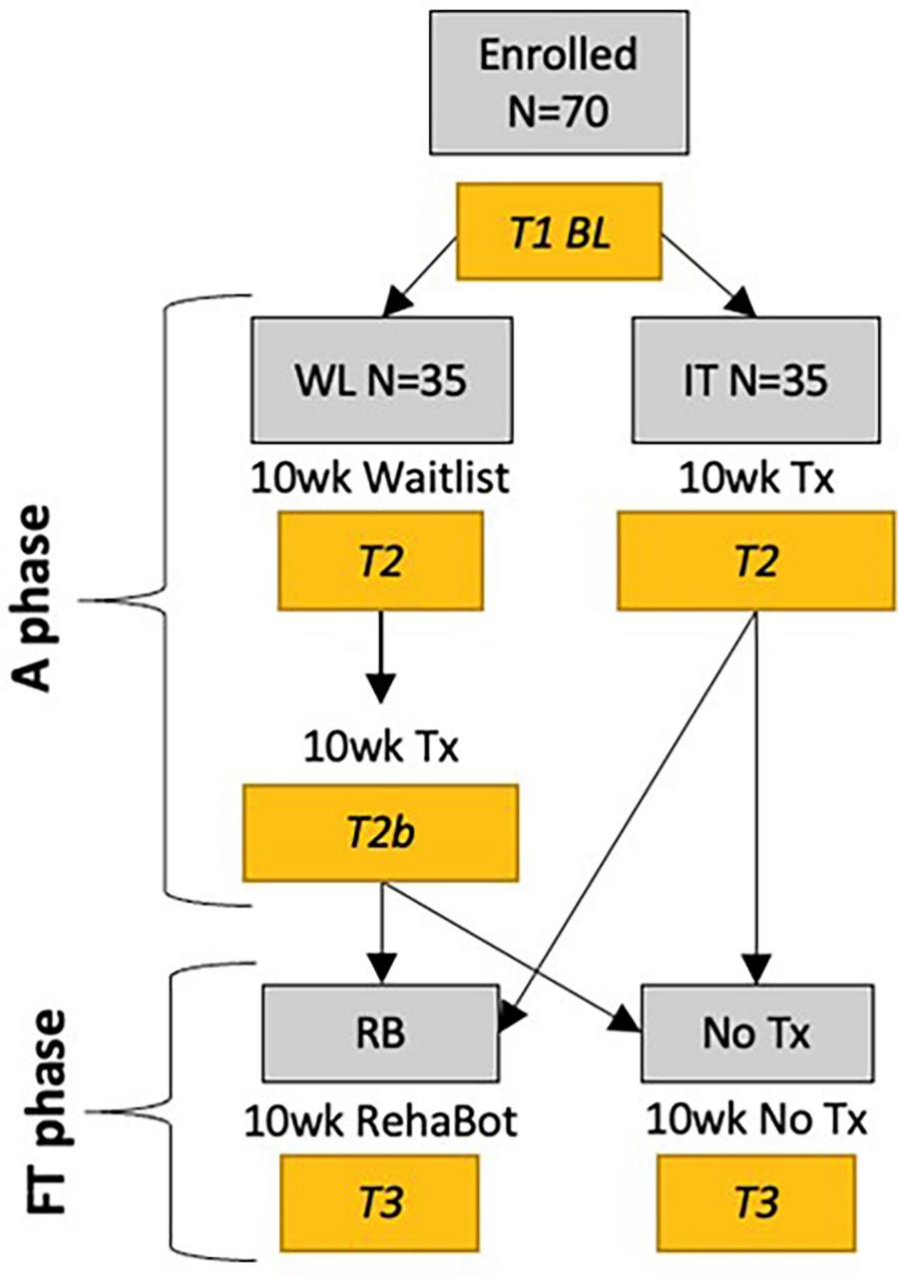
Schematic of trial design showing acquisition (A) and follow-through (FT) phases. BL, baseline; WL, waitlist; IT, immediate treatment; Tx, treatment.

This is a randomized waitlist-controlled trial, registered prior to participant recruitment at https://www.clinicaltrials.gov (NCT06028334). As shown in Figure 1, after a baseline assessment, participants are randomized 1:1 into IT (10 weeks of GetUp&Go intervention) or WL (10 weeks on waitlist), after which the primary outcome is measured. WL participants then receive 10 weeks of GetUp&Go. The 10-week treatment period (for all participants) is termed the Acquisition or A phase. Directly after the A phase, participants are re-randomized 1:1 into the Follow-Through (FT) phase: 10 additional weeks, of either RB or No Tx. A final outcome assessment and a debriefing interview are conducted at the end of the FT phase.

### 2.2 Participants

Participants will be 70 persons who meet the following inclusion criteria: (1) age ≥18; (2) TBI (open or closed), sustained at least 6 months prior to enrollment, of at least moderate severity as evidenced by: (a) loss or alteration of consciousness ≥30 min and/ or post-traumatic amnesia (PTA) ≥ 24 h, not due to intoxication/ sedation *and* documented prospectively from the injury (i.e., not retrospectively self-reported); and/ or (b) positive neuroimaging findings consistent with TBI; (3) fully weight bearing on lower limbs and able to walk indoors and outdoors without the assistance of another person (independent use of an assistive device such as a cane is acceptable); (4) cognitively able to participate in treatment, using selected items from the Glasgow Outcome Scale-Extended (49); (5) able to communicate adequately in English for participation in the treatment protocols; (6) informed consent given by participant. Participants are excluded for (1) contraindications to increasing PA as judged by a study physician and/ or study Physical Therapist, using an exam adapted from published screening tools (50); (2) medical or psychiatric instability, including current psychosis or severe uncontrolled substance misuse, as assessed using items from the Mini-International Neuropsychiatric Interview (51), or suicidal ideation with intent or plan, as assessed by the Columbia-Suicide Severity Rating Scale, screening version (52); (3) significant physical or intellectual disability predating the TBI; (4) neurodegenerative disorder, e.g., Parkinson's disease, dementia; (5) insufficiently inactive, i.e., reporting >23 weekly moderate/ vigorous activity units on the Godin Leisure-Time Exercise Questionnaire (53); (6) planned surgery or other hospitalization during the succeeding 9 months; (7) physical or sensory disability (e.g., blindness; severe bimanual incoordination) that prevents use of a smartphone.

### 2.3 Measures

The measures collected at each time point are displayed in Table 1. The baseline evaluation (T1) includes psychometric measures and questionnaires as well as demographic and injury-related information collected from medical records and participant interview. Because we have found that primary medical chart information is variable for people with chronic TBI, we measure PTA duration with a structured interview that has been used in other studies and that correlates well with prospectively measured PTA (54). The primary outcome (average activity counts/ minute) and other objective PA outcomes are measured using the wrist-worn Actigraph GT3XP. Activity counts using the GT3XP correlate well with biometric measures of energy expenditure (55). Following a practice prevalent in the PA measurement literature (56, 57), daily activity counts are averaged only from a minimum of 4 full wear days out of each 7-day wear period; if 4 days of data are not recorded, the wear period is repeated or discarded.

**Table 1:** Measures administered at each data collection point.

Type and name of measure	T1	T2/T2b	T3
Demographics: Age, race/ ethnicity, education	X		
Injury severity (estimated days of PTA), time post injury	X		
Episodic memory: Rey Auditory Verbal Learning Test (58) z-scores	X		
Executive function: Trail Making Test (59), Brixton Spatial Anticipation Test (60)	X		
Speed of processing: Symbol Digit Modalities Test (61)	X		
Conscientiousness/ other personality factors: NEO Personality Inventory-Revised (62)	X		
Living situation; use of walking aid; Body Mass Index	X	X	X
Neighborhood characteristics: Safety, walkability	X	X	X
Social support for PA (63, 64)	X	X	X
Type and level of motivation for PA: Exercise Regulations Questionnaire (BREQ-3) (65)	X	X	X
Self-efficacy, outcome expectancies, and identity/ values related to PA (64, 66–68)	X	X	X
Outcome measures			
Accelerometer: average counts/ minute from 4 + days over 7-day wearing period (primary outcome)	X	X	X
Accelerometer: % time sedentary, % time engaged in MVPA, avg daily step count	X	X	X
Self-reported PA: Godin Leisure-Time Exercise Questionnaire (53)	X	X	X
Emotional function: Brief Symptom Inventory-18 (69)	X	X	X
Fatigue: Fatigue Severity Scale, short form (70)	X	X	X
Sleep quality: Pittsburgh Sleep Quality Index (71)	X	X	X
Pain: PROMIS Pain Interference Scale, short form 6b (72)	X	X	X
Health-related quality of life: Quality of Life After Brain Injury (73)	X	X	X

### 2.4 Procedure

This study is approved and overseen by an Institutional Review Board (iRISID-2023-1533) and complies in full with the Helsinki Declaration for the protection of human subjects.

Participants are recruited from a variety of sources including two research registries at the study site, as well as clinical programs at the site and others in the geographical vicinity. Recruitment began in December, 2023 and is expected to continue until December, 2026. Prospective participants are screened for eligibility over the phone after expressing interest in the study and assenting to screening. Eligible participants are invited to an in-person session that includes written consent, administration of T1 measures listed in Table 1, and screening by both the study physician and the study Physical Therapist. These screenings include a review of systems and assessment of vital signs, lower extremity range of motion, and mobility. Exercise tolerance is evaluated using the modified Bruce protocol (74). Following the physical/ medical screening and the T1 evaluation, participants are given the Actigraph and instructed to wear it continuously for 7 days as they go about their normal routines in the home and community. Participants are given postage-paid padded envelopes for returning the devices.

After all baseline data including the Actigraph data are collected, participants are randomized 1:1 to IT or WL. Randomization uses a sequence of permuted blocks randomly selected from sizes 6, 9, and 12. The randomization sequence was created by the study statistician and is kept in a securely housed spreadsheet which kept all but the current treatment assignment obscured from view. To maximize equipoise and minimize attrition, participants are informed that the randomization is to “one baseline evaluation” or “two baseline evaluations 10 weeks apart.” WL participants receive periodic contacts from research staff reminding them of their upcoming activities in the program. After this initial 10-week period, the primary outcome is measured with another 7-day Actigraph wear period, followed by telephone administration of the outcome measures listed in Table 1.

When all post-treatment data are collected (at T2 for IT and T2b for WL), participants are re-randomized 1:1 to 10 weeks of RB or No Tx (see Figure 1). T3 data are collected after that interval and as a final step, the participant receives a 15-minute debriefing interview by phone. This interview includes the Patient Global Impression of Change scale (75), worded as follows: “Since your participation in this study, how would you describe the change (if any) in areas of your life that could be related to physical activity and fitness, such as physical and mental well-being, mood, and overall quality of life?” Responses are on a 7-point scale from “No change (or condition has gotten worse)” to “A great deal better and a considerable improvement that has made all the difference.” Other questions are concerned with features of the treatment and the RehaBot app that the participant particularly liked or disliked.

This trial uses masked outcome assessment, with special precautions taken to prevent inadvertent unmasking. These include making treatment information completely inaccessible to data collection staff, and using a “script” reminding participants not to discuss any of their experiences in the program when contacted by a data collector. Any instances of inadvertent unmasking are recorded for later analysis of their influence, and data collectors are asked to guess the participant's treatment allocation following each assessment.

Adverse events (AEs) are documented systematically throughout the trial. AEs are defined as any of the following: 1) Events that initiate the risk management protocol (i.e., expression of suicidal ideation or intent), 2) Events prompting an unscheduled emergency department visit not resulting in hospitalization, 3) Any unanticipated event that is unexpected and related to the study treatment. Serious Adverse Events (SAEs) are defined as outcomes resulting in death, hospitalization, life-threatening circumstances, person at risk of death at the time of the event, disability and/or incapacity, or events requiring intervention to prevent these outcomes. All hospitalizations are considered SAEs, with the exception of planned hospitalizations for elective procedures unrelated to the study intervention. AEs and SAEs are tracked through multiple surveillance mechanisms: spontaneous reporting by participants, discovery by study staff, identification by clinicians during therapy sessions, or detection during study assessments. When an AE is identified or reported, the therapist/data collector completes an Adverse Event Form immediately. This form documents the adverse event type, duration and timing, event details, and resolution status. AEs are reviewed periodically by a Data Safety Monitoring (DSM) team consisting of a physiatrist with extensive experience in msTBI research, and a statistician with expertise in clinical trials methodology. The DSM team also reviews and makes recommendations on recruitment, attrition, and other data relevant to the conduct of the trial and human subjects protections.

### 2.5 Intervention

The overall goal of the intervention is to develop and support a personalized plan to increase physical activity and decrease sedentary behavior, using treatment ingredients consonant with the COM-B model (27). The ingredients in each COM-B domain (Capability, Opportunity, Motivation) that are supplied by the therapist and by RehaBot are summarized in Table 2.

**Table 2:** Treatment ingredients in theoretically defined domains in the GetUp&Go program. Ingredients delivered via RehaBot are *italicized.*

COM-B domain	Treatment Ingredients
Capability: Physical	-Elicit past skills/ experiences and current preferences re: PA -Steer P toward safe/ appropriate activities as per MD/ PT screening; provide instruction for safe/ effective PA as needed
Capability: Psychological/ Cognitive	-Education about PA: benefits, types, dose-response relationship -Provide menus and ideas for PA at all levels of intensity; indoors and outdoors; scheduled bouts and incidental activity -Teach how to monitor exertion level, as needed -Provide structure and prompts for action planning (where, when, how, how often, with whom PA will be performed) and coping planning (how to deal with anticipated obstacles to PA) -*Provide reminders of planned activities in P's daily environment* -*Provide on-demand support by reminding P of daily plans and coping plans*
Opportunity: Social	-Elicit information about familial and sociocultural norms/ factors that may affect P's pursuit of PA -Encourage P to solicit support and/ or co-participation in PA from family and friends
Opportunity: Physical	-Assess/ discuss perceived neighborhood characteristics (walkability, aesthetics, safety, amenities) -Assess/ discuss home/ lifestyle affordances (space, time); assist with time management as needed -Elicit information, problem-solve, and provide tips regarding equipment/ clothing, facilities, transportation, costs, other resources -Encourage PA in varied contexts to promote generalization
Motivation: Reflective	-Assist P in formulating/ stating macro goals and intentions regarding PA; prompt/ discuss self-assessment of commitment level -Assess self-efficacy regarding chosen PA, ability to cope with obstacles, and ability to schedule PA in daily life; provide feedback and information to enhance self-efficacy; remind P of past and current successes -Assess for, encourage, and reinforce autonomous motivation for PA -Elicit expected positive outcomes; elicit and educate re: negative beliefs -Teach/ encourage motivational self-talk, including implementation intentions -Provide verbal reinforcement and feedback on gains -*Support self-monitoring by prompting P to report on progress towards goals and positive outcomes/ satisfaction with increased PA in their daily environment as they complete activities* -*Provide reinforcing messages in the moment as P completes activities* -*Provide on-demand feedback on accomplishments and progress towards goals* -*Provide on-demand support by reminding P of macro goal intentions regarding PA*
Motivation: Automatic	-Explore P's implicit associations with PA as pleasant/ unpleasant -Steer P toward activities that evoke pleasure, enjoyment -Explore and encourage P's values related to PA and self-identification as an (e.g.) active person/ fit person/ role model to others -Prompt P to use habit formation strategies such as distinctive activity-initiation cues in environment, time-of-day cues -*Assist in habit formation by cuing P's activities in daily environment*

P, participant; MD, medical doctor; PT, Physical Therapist; PA, physical activity.

The 10-week GetUp&Go intervention is delivered entirely remotely. The first 2 sessions last 60–90 min each and are delivered within the first week over Zoom for Healthcare (or smartphone if needed). The following 3 sessions (in weeks 3, 5, and 8) are delivered by phone and last 30–45 min. The RehaBot app is installed on the participant's phone in Session 2 followed by a guided overview and brief training that includes hands-on practice, troubleshooting, and opportunities to ask questions, until the participant expresses confidence in using the app. Participants who do not own a smartphone or do not have access to a computer/ tablet are issued a phone with unlimited texting and/ or an iPad for the duration of the trial. To maximize scheduling flexibility, participants are assigned to one of 4 therapists: Two doctoral level neuropsychologists, one Masters-trained clinician with extensive experience in neuropsychological approaches to the treatment of msTBI, and one doctoral-level Physical Therapist. All therapists are experienced clinicians with specific expertise in treating persons with msTBI. To ensure fidelity, a detailed manual is used for all treatment sessions. In addition, two therapists were present during treatment sessions for the first four participants in the trial in order to achieve proficiency and consistency in delivering the intervention. All therapists continue to meet weekly to review cases to ensure that their approaches and procedures are comparable.

**Figure 2:**
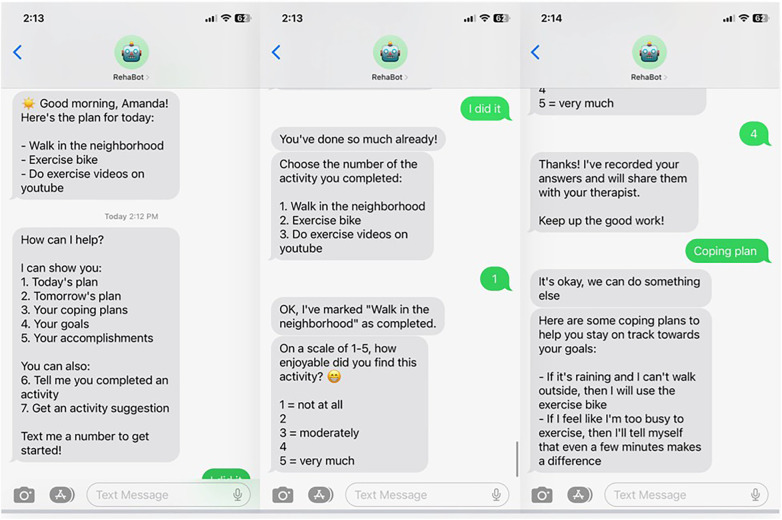
Example of RehaBot screens during interaction with participant.

In Session 1, the therapist provides education on the benefits of PA, emphasizing the dose-response relationship (i.e., the fact that *any* degree of PA increase is potentially beneficial). In discussion with the participant, the therapist explores in more depth the factors revealed in screening, and they begin to brainstorm a list of possible activities and routines to increase PA/ decrease sedentary behavior within the participant's preferences and opportunities in the home and community environment. The therapist promotes consideration of new activities, existing activities that might be modified or increased, and ways to “sneak in” PA, such as taking extra steps to reach a destination. The therapist also elicits overall goals and specific intentions regarding increased PA and reduction of sedentary time (64, 76), expected outcomes (64), and other statements for later use as personalized motivational reminders, delivered by RehaBot. The capabilities of RehaBot are explained and demonstrated in Session 1 using sample screen shots, to help participants begin to consider how they might benefit from using the app. See Figure 2 for sample screenshots.

As the list of planned activities takes shape, the therapist engages the participant in action planning, i.e., specific plans for when, where, how often, and (if applicable) with whom activities will occur during the week (64, 76–78). Additional discussion involves potential barriers to activity completion, and possible ways of overcoming them. Obstacles may be overcome with various types of coping plans for one-time solutions (e.g., buying suitable shoes for walking or jogging; joining the nearby fitness center), or via just-in-time reminders framed as implementation intentions (76, 79, 80) (e.g., “If I don't feel like taking my walk, I’ll remind myself that….”) that may be accessed through RehaBot.

In Session 2, RehaBot is installed on the participant's phone, the participant is trained on all of its features, and the therapist and participant collaborate on personalizing the app. RehaBot is accessed via regular text-messaging, and engages in bi-directional communication with the user (see Figure 2). Therapists use a portal to enter participants' action plans, coping plans, goals, and other relevant data into the RehaBot app. Message timing is fully customizable. Participants select their preferred times for daily “good morning” and “good evening” check-in messages. They also determine how many activity reminder messages they wish to receive throughout the day and when those reminders should be delivered. Scheduled contacts come in the form of text message reminders to check your plan for the day, carry out a planned activity at a scheduled time, and report on your accomplishments. However, RehaBot is also available on-demand to serve as a “coach” to provide reminders, suggestions, feedback, reinforcement, coping plans, and personalized motivational messages. Activity reminder messages are worded using the participant's own language and selected activities. Participants also personalize the content of activity suggestions and the language and content of their coping plans. When an activity is logged as complete, RehaBot sends encouraging messages that reinforce the effort and tie the participant's accomplishments to progress towards a specific personal goal. Self-efficacy, thought to be a strong mediator of positive behavior change, is a particular target of intervention via specific positive feedback from both therapist and chatbot, and reminders of task mastery and overall progress (81).

In the phone sessions in weeks 3, 5, and 8, the participant's plan and their reactions to it are reviewed. Progress is reinforced, problems and obstacles are addressed, and any desired changes to the plan are instituted in the RehaBot portal. In the final telephone session, the therapist guides the participant in reflecting on their overall progress and their ability to overcome barriers to physical activity, while offering feedback on positive changes observed throughout the intervention. The participant continues to use RehaBot independently until Week 10, when they receive a letter reminding them of the upcoming telephone data collection and Actigraph wear period.

Participants randomized to RB for the FT phase receive a call notifying them that their RehaBot plan will remain in place for another 10 weeks. These participants are encouraged to use RehaBot to continue with their activity plans until the next evaluation period. Those randomized to No Tx are reminded of the final evaluation in 10 weeks and encouraged to keep up their PA plans. Neither FT condition includes any further contact from the therapist, although we would respond to participants who contacted us regarding a technical problem with the app.

### 2.6 Data analysis

For the primary endpoint (*Hypothesis 1a*: **primary hypothesis**) and each of the secondary endpoints (*Hypothesis 1b*), the mean change from T1 to T2 in the IT group will be compared to the mean change from T1 to T2 in the WL group using the two-sided two-sample t-test with alpha = 0.05. The mean change in each group and the mean difference between changes in IT vs. WL group will be estimated with the corresponding 95% confidence interval. If the normal distribution assumption is not appropriate for patient-specific changes in one or both groups, then the two-sided two-sample Wilcoxon test will be used instead of the t-test. We will employ intent-to-treat for the *primary analysis*, and as such, we will make every attempt to collect T2 outcome data on all randomized participants regardless of treatment received. Per current recommendations (82–84), we will use multiple imputation to account for missing T2 data, and evaluate missingness assumptions (by comparing available data for participants with complete vs. missing T2). If the assumption of the data being *missing at random* is not appropriate, the imputations will be performed using the methods specifically developed for data *missing not at random* (85). As a *secondary analysis* we will perform a complete-case analysis with dropouts excluded. With the proposed sample size of 35 subjects per arm and assuming up to 15% loss to follow-up, we expect to have at least 30 subjects per arm available for analysis. The sample size of 30 subjects per arm provides 81% power to detect the mean difference between changes in IT vs. WL arms corresponding to the effect size of 0.75 (effect size for the primary endpoint as reported in Clanchy et al. (24) using the two-sided two-sample t-test with alpha 0.05.

For *Hypothesis 2a*, the primary endpoint is the change in average accelerometer activity counts/ day from post-intervention timepoint (T2 for IT participants, T2b for WL) to the follow-up endpoint, T3, 10 weeks later. The subject-specific T2/T2b-T3 changes will be modeled in an ANCOVA model as dependent on group (RB vs. No Tx) and the average accelerometer activity counts/day during the A phase (from timepoint T1 to post-intervention timepoint T2/T2b). The interaction between group and T1-T2(b) changes will be considered and retained in the model, if significant. If the interaction is not significant, Hypothesis 2a will be tested by comparing the mean change in RB vs. No Tx group using the model-based two-sided two-sample t-test with alpha 0.05. In the case of the main effects model (no significant interaction), this is equivalent to testing the main effect of RB (vs. No Tx) as the difference between intercepts of the regression lines with T1-T2(b) change as a predictor. A significant interaction would imply that the effect of RB (vs. No Tx) depends on the amount of change achieved during the A phase, and the test of interaction corresponds to comparing the slopes of the T1-T2(b) change regression lines between RB and No Tx groups. In the case of a significant interaction, the difference between RB and No Tx groups will be quantified in terms of the difference between the T1-T2(b) change regression slopes, and the significance (*p*-value) for that difference is the same as the significance (*p*-value) of the interaction. It is not expected that this interaction is important, and the study is not powered to detect such interaction. The secondary outcome measures (*Hypothesis 2b)* will be analyzed using the same approach.

We will report AEs and SAEs descriptively by treatment arm, including the number and percentage of participants experiencing at least one AE/SAE, as well as the total number of events by category. All AEs will be categorized by severity, relatedness to the intervention, and resolution status.

With regard to power, in the study of Clanchy et al. (24), participants in the active intervention group lost a mean of 1.7 standard deviations in average activity counts per day between their post-treatment and follow-up assessments, without any treatment during this time (comparable to our No Tx condition). We hypothesize that the loss in activity in the RB (treated) group in our study will be reduced by at least 50% of this value, an expected difference in effect size between RB and No Tx groups of 0.85. We make the conservative assumption of up to 15% additional attrition during the FT phase, leaving *N* = 46. With this sample size we will have 80% power to detect a difference in effect size of at least.85 between groups, using the two-sided two-sample t-test with alpha 0.05.

The analyses of predictors of treatment response will be exploratory, as the large number of predictors of possible interest will preclude hypothesis-driven methods. We will consider the use of analysis techniques such as model averaging (86, 87)*,* a method that allows for more robust conclusions about important candidate predictors when the possible predictors are numerous and the sample is relatively small, and when different variable selection methods may result in different and possibly suboptimal models.

## 3 Anticipated results

In this manuscript we describe a randomized controlled trial of a novel intervention intended to promote PA in individuals with chronic msTBI, a population at risk for reduced PA and increased sedentary behavior. The GetUp&Go intervention includes a combination of features that are unique in the msTBI literature to date: Treatment ingredients that are based in a strong theoretical model and vetted by people with lived experience of msTBI; a standardized treatment manual that also allows for individualized PA plans; remote treatment delivery, with a chatbot to supplement and support increased PA; and objectively measured PA as a primary outcome, with a wide variety of secondary outcome measures for assessment of the broader impact of the intervention. In addition, the post-treatment follow-up phase is designed to evaluate the impact of continued chatbot support on maintenance of treatment gains. If use of the chatbot results in less diminution of gains, as we have hypothesized, this could offer one type of solution to the problem noted in prior studies: that participants with msTBI tend not to persist with successful PA programs once therapeutic support is withdrawn.

Whether or not the improvements in PA and the secondary outcomes occur as hypothesized, we anticipate that our exploratory analyses will provide valuable information on the predictive factors of treatment success. This information, enhanced with the qualitative data supplied in debriefing interviews of study participants, will help enable the refinement of the protocol for future research.

### 3.1 Limitations

The sample population for this trial is limited to persons with chronic msTBI who are living in the community, and who are able to walk without the assistance of another person. Our findings may not generalize to those with mild or acute TBI, institutionalized people, or those who are non-ambulatory.

## 4 Discussion

The conclusions from this trial must, of course, await the completion of data collection and analysis. However, we believe that PA enhancement provides a valuable and under-studied direction for msTBI. People with chronic msTBI experience a variety of challenges to both mental and physical well-being; and there is unassailable evidence that increasing PA leads to a broad array of improvements in both physical and mental health. The dose-response relationship between PA increase and health benefit suggests that people who are unable to achieve published guidelines for moderate-vigorous PA may still enjoy the fruits of more modest efforts. It is known that PA is valued by people with msTBI and that they are interested in finding ways to become more active; and PA programs may readily be tailored to individual abilities, preferences, and social/ environmental circumstances. Moreover, treatment programs that incorporate theoretically motivated and empirically validated behavior change ingredients may help to circumvent difficulties with initiation, persistence, and memory that can interfere with the ability to develop and maintain enhanced PA habits and routines following msTBI.

Regardless of outcome, this trial will advance the field of neurorehabilitation by addressing a critical gap identified in prior research: the lack of RCTs of theoretically motivated interventions to promote PA for those with msTBI. The GetUp&Go intervention incorporates evidence-based behavior change techniques shown to be effective in other populations, while systematically adapting them to address the specific cognitive, emotional, and physical challenges faced by people with chronic msTBI. In addition, this trial will generate critical feasibility data for delivering behavioral interventions remotely to people with msTBI—a population that faces significant barriers to accessing in-person rehabilitation services due to transportation limitations, cognitive and physical problems, and geographic distance from specialized providers. The integration of mobile health technology represents an innovative approach to overcoming these barriers. Our findings relevant to feasibility, adherence, and user experience will provide valuable insights for refining remote delivery approaches, further adding to the evidence for telehealth interventions for people with chronic msTBI (88).

## 5 Recommendations

The design of this trial illustrates several features that are empirically supported yet too little employed in studies of msTBI rehabilitation. For future trials, we encourage the use of well-studied treatment ingredients that are known to enhance behavior change in the domain of physical activity. We also recommend continued exploration of remote interventions, particularly those capitalizing on SMS and mobile apps with demonstrated feasibility and acceptability in this population. Finally, we strongly recommend the inclusion of persons with lived experience of msTBI in the design and implementation of all treatment studies.

## Data Availability

The original contributions presented in the study are included in the article/Supplementary Material, further inquiries can be directed to the corresponding author.
